# Supplementary material for: Healthy decisions in the cued-attribute food choice paradigm have high test-retest reliability
Source: Sci Rep. 2021 Jun 18;11:12844. doi: 10.1038/s41598-021-91933-6 (PMC8213742; doi:10.1038/s41598-021-91933-6)
Supplement: Supplementary file 1 — Supplementary Information. [file 41598_2021_91933_MOESM1_ESM.pdf]

## Supplementary Information

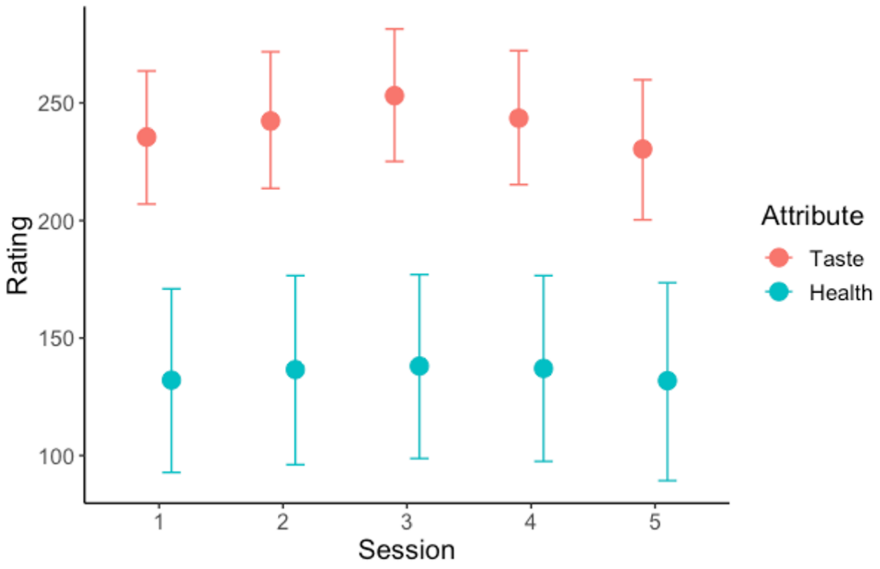


**Figure S1. Ratings over sessions.** The ratings didn't significantly changed over sessions. The error bars indicate standard error of means.

| Parameter | Mean | SD | 2.5% | 97.5% |
|---|---|---|---|---|
| Intercept | 0.20 | 0.13 | -0.06 | 0.46 |
| td | 1.22 | 0.14 | 0.94 | 1.51 |
| hd | -0.02 | 0.09 | -0.20 | 0.15 |
| condition1 | 1.52 | 0.24 | 1.04 | 1.98 |
| session2 | 0.28 | 0.09 | 0.10 | 0.46 |
| session3 | 0.07 | 0.08 | -0.09 | 0.23 |
| session4 | 0.11 | 0.10 | -0.08 | 0.30 |
| session5 | 0.05 | 0.09 | -0.13 | 0.23 |
| td:condition1 | -0.73 | 0.15 | -1.02 | -0.45 |
| hd:condition1 | 1.22 | 0.19 | 0.85 | 1.60 |
| td:session2 | -0.17 | 0.09 | -0.35 | 0.02 |
| td:session3 | -0.16 | 0.08 | -0.32 | 0.00 |
| td:session4 | -0.21 | 0.09 | -0.40 | -0.03 |
| td:session5 | -0.09 | 0.14 | -0.36 | 0.17 |
| hd:session2 | 0.04 | 0.08 | -0.13 | 0.21 |
| hd:session3 | 0.00 | 0.08 | -0.15 | 0.15 |
| hd:session4 | 0.04 | 0.08 | -0.13 | 0.20 |
| hd:session5 | 0.10 | 0.08 | -0.06 | 0.27 |
| condition1:session2 | -0.39 | 0.13 | -0.65 | -0.12 |
| condition1:session3 | -0.29 | 0.12 | -0.53 | -0.05 |
| condition1:session4 | -0.64 | 0.12 | -0.88 | -0.41 |
| condition1:session5 | -0.25 | 0.13 | -0.49 | 0.00 |
| td:condition1:session2 | 0.16 | 0.11 | -0.06 | 0.38 |
| td:condition1:session3 | 0.14 | 0.11 | -0.07 | 0.35 |
| td:condition1:session4 | 0.20 | 0.11 | -0.01 | 0.41 |
| td:condition1:session5 | 0.20 | 0.12 | -0.03 | 0.42 |
| hd:condition1:session2 | -0.30 | 0.13 | -0.56 | -0.03 |
| hd:condition1:session3 | -0.33 | 0.12 | -0.57 | -0.09 |
| hd:condition1:session4 | -0.54 | 0.12 | -0.77 | -0.31 |
| hd:condition1:session5 | -0.42 | 0.13 | -0.67 | -0.17 |

**Table S1.** Healthier Choice Logistic Regression Model. This table lists all main effects and interactions for the Hierarchical Bayesian logistic regression in Equation 1 (see Methods section). The columns labeled 2.5% and 97.5% indicate the lower and upper bounds of the 95% highest density interval for each parameter, respectively. SD = standard deviation.

| Parameter | Mean | SD | 2.5% | 97.5% |
|---|---|---|---|---|
| Intercept | 0.13 | 0.01 | 0.1 | 0.16 |
| td | -0.02 | 0.01 | -0.03 | -0.01 |
| hd | 0 | 0 | -0.01 | 0.01 |
| condition1 | -0.02 | 0.01 | -0.03 | 0 |
| session2 | 0 | 0.01 | -0.02 | 0.01 |
| session3 | -0.03 | 0.01 | -0.05 | -0.01 |
| session4 | -0.04 | 0.01 | -0.07 | -0.02 |
| session5 | -0.04 | 0.01 | -0.07 | -0.01 |
| td:condition1 | 0 | 0.01 | -0.01 | 0.02 |
| hd:condition1 | -0.03 | 0.01 | -0.05 | -0.02 |
| td:session2 | 0.01 | 0.01 | 0 | 0.02 |
| td:session3 | 0.01 | 0.01 | 0 | 0.02 |
| td:session4 | 0 | 0.01 | -0.01 | 0.01 |
| td:session5 | 0 | 0.01 | -0.01 | 0.01 |
| hd:session2 | 0 | 0.01 | -0.01 | 0.01 |
| hd:session3 | -0.01 | 0 | -0.02 | 0 |
| hd:session4 | -0.01 | 0 | -0.02 | 0 |
| hd:session5 | -0.01 | 0.01 | -0.02 | 0 |
| condition1:session2 | -0.01 | 0.01 | -0.03 | 0.01 |
| condition1:session3 | 0 | 0.01 | -0.02 | 0.02 |
| condition1:session4 | 0 | 0.01 | -0.02 | 0.02 |
| condition1:session5 | -0.01 | 0.01 | -0.03 | 0.01 |
| td:condition1:session2 | 0 | 0.01 | -0.02 | 0.01 |
| td:condition1:session3 | 0 | 0.01 | -0.02 | 0.01 |
| td:condition1:session4 | 0.01 | 0.01 | -0.01 | 0.02 |
| td:condition1:session5 | 0.01 | 0.01 | 0 | 0.03 |
| hd:condition1:session2 | 0.01 | 0.01 | 0 | 0.02 |
| hd:condition1:session3 | 0.01 | 0.01 | 0 | 0.03 |
| hd:condition1:session4 | 0.02 | 0.01 | 0 | 0.03 |
| hd:condition1:session5 | 0.01 | 0.01 | 0 | 0.02 |

**Table S2.** RT Linear Regression Model, Part I. This table lists all main effects and interactions from the linear regression in Equation 2 (see Methods section) for non-challenge trials. The main effect of challenge trials and its interactions are reported in S3 on the next page for the sake of space. Note that challenge trials are defined as those in which the tastier item is not also the healthier item (i.e. there is a conflict between the taste and health attributes). The regression coefficients in this table represent effects on log(RT) in the subset of trials in which taste and health attributes are aligned. The columns labeled 2.5% and 97.5% indicate the lower and upper bounds of the 95% highest density interval for each parameter, respectively. SD = standard deviation.

| Parameter | Mean | SD | 2.5% | 97.5% |
|---|---|---|---|---|
| challenge1 | 0.03 | 0.01 | 0.01 | 0.05 |
| td:challenge1 | 0.06 | 0.01 | 0.04 | 0.08 |
| hd:challenge1 | -0.01 | 0.01 | -0.02 | 0 |
| condition1:challenge1 | -0.02 | 0.01 | -0.04 | 0.01 |
| session2:challenge1 | 0 | 0.01 | -0.03 | 0.02 |
| session3:challenge1 | -0.02 | 0.01 | -0.04 | 0.01 |
| session4:challenge1 | -0.01 | 0.01 | -0.03 | 0.02 |
| session5:challenge1 | -0.02 | 0.01 | -0.05 | 0.01 |
| td:condition1:challenge1 | -0.04 | 0.01 | -0.06 | -0.02 |
| hd:condition1:challenge1 | 0.02 | 0.01 | 0 | 0.03 |
| td:session2:challenge1 | -0.02 | 0.01 | -0.04 | 0 |
| td:session3:challenge1 | -0.02 | 0.01 | -0.04 | 0 |
| td:session4:challenge1 | -0.01 | 0.01 | -0.03 | 0.01 |
| td:session5:challenge1 | 0 | 0.01 | -0.02 | 0.02 |
| hd:session2:challenge1 | 0.01 | 0.01 | -0.01 | 0.03 |
| hd:session3:challenge1 | 0 | 0.01 | -0.01 | 0.02 |
| hd:session4:challenge1 | 0 | 0.01 | -0.02 | 0.01 |
| hd:session5:challenge1 | 0 | 0.01 | -0.01 | 0.02 |
| condition1:session2:challenge1 | 0 | 0.02 | -0.04 | 0.03 |
| condition1:session3:challenge1 | 0.02 | 0.02 | -0.01 | 0.06 |
| condition1:session4:challenge1 | 0 | 0.02 | -0.03 | 0.04 |
| condition1:session5:challenge1 | 0.01 | 0.02 | -0.03 | 0.05 |
| td:condition1:session2:challenge1 | 0.02 | 0.01 | -0.01 | 0.04 |
| td:condition1:session3:challenge1 | 0.03 | 0.01 | 0 | 0.05 |
| td:condition1:session4:challenge1 | 0 | 0.01 | -0.02 | 0.03 |
| td:condition1:session5:challenge1 | -0.01 | 0.01 | -0.04 | 0.02 |
| hd:condition1:session2:challenge1 | -0.01 | 0.01 | -0.04 | 0.01 |
| hd:condition1:session3:challenge1 | 0 | 0.01 | -0.02 | 0.02 |
| hd:condition1:session4:challenge1 | 0.01 | 0.01 | -0.02 | 0.03 |
| hd:condition1:session5:challenge1 | 0 | 0.01 | -0.02 | 0.03 |

**Table S3.** RT Logistic Regression, Part II. This table lists the main effect of challenge trials and its interactions from the linear regression in Equation 2 (see Methods section). Challenge trials are defined as those in which the tastier item is not also the healthier item (i.e. there is a conflict between the taste and health attributes). Thus, the regression coefficients in this table represent effects on log(RT) in the subset of trials in which taste and health attributes are in conflict with regard to which option is preferred. The columns labeled 2.5% and 97.5% indicate the lower and upper bounds of the 95% highest density interval for each parameter, respectively. SD = standard deviation.

| Parameter | Mean | SD | 2.5% | 97.5% |
|---|---|---|---|---|
| Intercept | 0.72 | 0.03 | 0.66 | 0.77 |
| condition1 | -0.15 | 0.03 | -0.21 | -0.1 |
| session2 | -0.02 | 0.03 | -0.08 | 0.03 |
| session3 | -0.02 | 0.03 | -0.08 | 0.03 |
| session4 | -0.03 | 0.03 | -0.08 | 0.03 |
| session5 | -0.01 | 0.03 | -0.06 | 0.05 |
| condition1:session2 | 0.02 | 0.04 | -0.06 | 0.1 |
| condition1:session3 | 0.03 | 0.04 | -0.05 | 0.1 |
| condition1:session4 | 0.04 | 0.04 | -0.04 | 0.12 |
| condition1:session5 | 0 | 0.04 | -0.08 | 0.08 |

**Table S4.** Hierarchical Bayesian regression of choice outcomes based on taste ratings. This table lists the main effect of condition and session and their interactions on whether participants choose the food items based on their subjective taste ratings (i.e., choose the item rated as more palatable). The ratings were made in session 1 and used to predict choices in sessions 1-5. The Intercept coefficient represents the probability of choosing the tastier option in natural trials during session 1. The condition1 effect is the difference between health-cued and natural trials in session 1. The main effects of session represent the difference between natural trials in sessions 2-5 versus 1. The condition1:session interactions represent the difference between health-cued and natural trials in sessions 2-5 versus 1. The columns labeled 2.5% and 97.5% indicate the lower and upper bounds of the 95% highest density interval for each parameter, respectively. SD = standard deviation.

| Parameter | Mean | SD | 2.5% | 97.5% |
|---|---|---|---|---|
| Intercept | 0.53 | 0.02 | 0.49 | 0.57 |
| condition1 | 0.06 | 0.02 | 0.02 | 0.11 |
| session2 | -0.03 | 0.02 | -0.07 | 0.01 |
| session3 | -0.02 | 0.02 | -0.06 | 0.02 |
| session4 | -0.01 | 0.02 | -0.05 | 0.03 |
| session5 | 0.01 | 0.02 | -0.03 | 0.05 |
| condition1:session2 | 0 | 0.03 | -0.06 | 0.06 |
| condition1:session3 | -0.01 | 0.03 | -0.07 | 0.04 |
| condition1:session4 | -0.03 | 0.03 | -0.09 | 0.02 |
| condition1:session5 | -0.03 | 0.03 | -0.09 | 0.02 |

**Table S5.** Hierarchical Bayesian regression of choice outcomes based on healthiness ratings. This table lists the main effect of condition and session and their interactions on whether participants choose the food items based on their subjective healthiness ratings (i.e., choose the item rated as healthier). The ratings were made in session 1 and used to predict choices in sessions 1-5. The Intercept coefficient represents the probability of choosing the tastier option in natural trials during session 1. The condition1 effect is the difference between health-cued and natural trials in session 1. The main effects of session represent the difference between natural trials in sessions 2-5 versus 1. The condition1:session interactions represent the difference between health-cued and natural trials in sessions 2-5 versus 1. The columns labeled 2.5% and 97.5% indicate the lower and upper bounds of the 95% highest density interval for each parameter, respectively. SD = standard deviation.

***ICC values per Subject***

| Subject | Sessions: {1,2,3,4,5} | | Sessions: {1,5} | |
|---|---|---|---|---|
| | **Health** | **Taste** | **Health** | **Taste** |
| **1** | 0.577745 | 0.831173 | 0.857825 | 0.655314 |
| **2** | 0.710789 | 0.785189 | 0.812352 | 0.64899 |
| **3** | 0.65895 | 0.939484 | 0.926619 | 0.646569 |
| **4** | 0.854535 | 0.912131 | 0.856691 | 0.779961 |
| **5** | 0.762729 | 0.916849 | 0.870482 | 0.694037 |
| **6** | 0.651713 | 0.900806 | 0.931706 | 0.603868 |
| **7** | 0.742485 | 0.943812 | 0.960971 | 0.757416 |
| **8** | 0.719123 | 0.848438 | 0.855465 | 0.595679 |
| **9** | 0.412589 | 0.83275 | 0.810879 | 0.346153 |
| **10** | 0.477552 | 0.870815 | 0.867848 | 0.526212 |
| **11** | 0.627396 | 0.785987 | 0.796585 | 0.619369 |
| **12** | 0.381176 | 0.744022 | 0.822111 | 0.376208 |
| **13** | 0.712292 | 0.834418 | 0.828867 | 0.688644 |
| **14** | 0.776159 | 0.909753 | 0.900005 | 0.659892 |
| **15** | 0.256022 | 0.656646 | 0.548468 | 0.272936 |
| **16** | 0.660534 | 0.929756 | 0.876701 | 0.471796 |
| **17** | 0.618747 | 0.847002 | 0.706359 | 0.489656 |
| **18** | 0.479577 | 0.924915 | 0.923731 | 0.496566 |
| **19** | 0.58999 | 0.842669 | 0.889275 | 0.556814 |
| **20** | 0.458402 | 0.732254 | 0.763743 | 0.573374 |
| **21** | 0.076062 | 0.110394 | -0.03161 | -0.07609 |
| **22** | 0.421588 | 0.70068 | 0.413519 | 0.301857 |
| **23** | 0.7 | 0.80698 | 0.726133 | 0.560946 |

**Table S6.** ICC values per subject for the subset of 60 food images rated in all 5 sessions/days (left) and the full set of 180 food images rated in sessions 1 and 5 (right).

***ICC values per food image across 5 sessions***

| Images | Health | Taste | Images | Health | Taste |
|---|---|---|---|---|---|
| **1** | 0.548981 | 0.360017 | **31** | 0.415038 | 0.374519 |
| **2** | 0.839613 | 0.678043 | **32** | 0.601435 | 0.510712 |
| **3** | 0.530135 | 0.435967 | **33** | 0.381346 | 0.500656 |
| **4** | 0.499733 | 0.524707 | **34** | 0.567766 | 0.599284 |
| **5** | 0.53434 | 0.570703 | **35** | 0.715889 | 0.812522 |
| **6** | 0.450081 | 0.623053 | **36** | 0.646967 | 0.600088 |
| **7** | 0.718801 | 0.671339 | **37** | 0.739166 | 0.641482 |
| **8** | 0.453639 | 0.597161 | **38** | 0.65807 | 0.70777 |
| **9** | 0.882878 | 0.594293 | **39** | 0.569736 | 0.664011 |
| **10** | 0.662751 | 0.51526 | **40** | 0.667879 | 0.656011 |
| **11** | 0.494073 | 0.709408 | **41** | 0.375358 | 0.564663 |
| **12** | 0.580517 | 0.716292 | **42** | 0.535164 | 0.432079 |
| **13** | 0.341531 | 0.412314 | **43** | 0.741156 | 0.538456 |
| **14** | 0.533415 | 0.618469 | **44** | 0.403696 | 0.384647 |
| **15** | 0.502964 | 0.394111 | **45** | 0.568851 | 0.431005 |
| **16** | 0.387244 | 0.533008 | **46** | 0.569612 | 0.561693 |
| **17** | 0.204656 | 0.397756 | **47** | 0.437377 | 0.585203 |
| **18** | 0.330214 | 0.433892 | **48** | 0.619319 | 0.648813 |
| **19** | 0.786993 | 0.66636 | **49** | 0.492178 | 0.58252 |
| **20** | 0.63245 | 0.681147 | **50** | 0.255104 | 0.533144 |
| **21** | 0.435499 | 0.568086 | **51** | 0.372384 | 0.506155 |
| **22** | 0.73386 | 0.656252 | **52** | 0.520931 | 0.599399 |
| **23** | 0.544993 | 0.486982 | **53** | 0.60342 | 0.570972 |
| **24** | 0.611825 | 0.669386 | **54** | 0.631005 | 0.699958 |
| **25** | 0.64508 | 0.658395 | **55** | 0.695773 | 0.711901 |
| **26** | 0.739376 | 0.596953 | **56** | 0.707249 | 0.678658 |
| **27** | 0.473611 | 0.770296 | **57** | 0.644811 | 0.740512 |
| **28** | 0.690062 | 0.491442 | **58** | 0.626182 | 0.719768 |
| **29** | 0.424566 | 0.710365 | **59** | 0.309981 | 0.519247 |
| **30** | 0.29652 | 0.170655 | **60** | 0.574235 | 0.492516 |

**Table S7.** ICC values per image across for the subset of 60 items rated in all five sessions.

***ICC values per image for sessions 1 and 5 only***

| image | health | taste | image | health | taste | image | health | taste | image | health | taste |
|---|---|---|---|---|---|---|---|---|---|---|---|
| **1** | 0.697576 | 0.471291 | **46** | 0.176739 | 0.629575 | **91** | 0.618157 | 0.65365 | **136** | 0.392833 | 0.74924 |
| **2** | 0.25823 | 0.783531 | **47** | -0.21208 | -0.05217 | **92** | 0.301997 | 0.337437 | **137** | 0.138446 | 0.324126 |
| **3** | 0.547048 | 0.666159 | **48** | -0.09588 | 0.022942 | **93** | 0.548629 | 0.319659 | **138** | 0.430071 | 0.841025 |
| **4** | 0.620692 | 0.63687 | **49** | 0.314135 | 0.418134 | **94** | -0.00697 | 0.400818 | **139** | 0.447608 | 0.441645 |
| **5** | 0.825931 | 0.562592 | **50** | 0.585291 | 0.651573 | **95** | 0.61529 | 0.699195 | **140** | 0.714605 | 0.567584 |
| **6** | 0.483205 | 0.292532 | **51** | 0.436907 | 0.627803 | **96** | 0.737655 | 0.613412 | **141** | 0.856409 | 0.720074 |
| **7** | 0.491307 | 0.15224 | **52** | 0.502873 | 0.438326 | **97** | 0.59231 | 0.522592 | **142** | 0.569853 | 0.452755 |
| **8** | 0.267829 | 0.232278 | **53** | 0.199694 | 0.497712 | **98** | 0.433851 | 0.52723 | **143** | 0.662252 | 0.154427 |
| **9** | 0.667372 | 0.566883 | **54** | 0.873442 | 0.517383 | **99** | 0.501924 | 0.277089 | **144** | 0.852084 | 0.566845 |
| **10** | 0.239501 | 0.622533 | **55** | 0.054421 | 0.346481 | **100** | 0.651407 | 0.715438 | **145** | 0.779361 | 0.450296 |
| **11** | 0.46995 | 0.424417 | **56** | 0.516997 | 0.811649 | **101** | 0.536671 | 0.479607 | **146** | 0.691585 | 0.732115 |
| **12** | 0.485187 | 0.529163 | **57** | 0.788941 | 0.248095 | **102** | 0.708827 | 0.75428 | **147** | 0.76623 | 0.678841 |
| **13** | 0.389698 | 0.78525 | **58** | 0.511837 | 0.163358 | **103** | 0.355003 | 0.42091 | **148** | 0.704897 | 0.41036 |
| **14** | 0.386874 | 0.770463 | **59** | 0.779937 | 0.742563 | **104** | 0.790134 | 0.559765 | **149** | 0.042335 | 0.284698 |
| **15** | 0.722393 | 0.829682 | **60** | 0.5763 | 0.786274 | **105** | 0.514148 | 0.799129 | **150** | 0.822873 | 0.655385 |
| **16** | 0.579377 | 0.664574 | **61** | 0.368137 | 0.572689 | **106** | 0.765182 | 0.647292 | **151** | 0.4449 | -0.04558 |
| **17** | 0.505108 | 0.758164 | **62** | 0.251526 | 0.461615 | **107** | 0.360294 | 0.661974 | **152** | 0.358764 | 0.719496 |
| **18** | 0.47395 | 0.622363 | **63** | 0.771498 | 0.787611 | **108** | 0.695452 | 0.374481 | **153** | 0.608609 | 0.329961 |
| **19** | 0.176377 | 0.558733 | **64** | 0.533722 | 0.530844 | **109** | 0.523211 | 0.603541 | **154** | 0.152314 | 0.192702 |
| **20** | 0.901769 | 0.716171 | **65** | 0.241014 | 0.501195 | **110** | 0.038523 | 0.357699 | **155** | 0.548745 | 0.610476 |
| **21** | 0.768926 | 0.239005 | **66** | 0.29474 | 0.744421 | **111** | 0.423804 | 0.079996 | **156** | 0.632847 | 0.51016 |
| **22** | 0.890165 | 0.246351 | **67** | 0.184351 | 0.378496 | **112** | 0.277337 | 0.710164 | **157** | 0.307929 | 0.570886 |
| **23** | 0.355194 | 0.610736 | **68** | 0.365805 | 0.460843 | **113** | 0.421327 | 0.579106 | **158** | 0.446877 | 0.688672 |
| **24** | 0.618139 | 0.534142 | **69** | 0.215326 | 0.570059 | **114** | 0.60044 | 0.441816 | **159** | 0.813133 | 0.325084 |
| **25** | 0.771925 | 0.380729 | **70** | 0.619867 | 0.717207 | **115** | 0.499749 | 0.617806 | **160** | 0.584323 | 0.784857 |
| **26** | 0.380696 | 0.542368 | **71** | 0.519222 | 0.38675 | **116** | 0.385659 | 0.567181 | **161** | 0.532151 | 0.617589 |
| **27** | 0.100362 | 0.152375 | **72** | 0.803613 | 0.739409 | **117** | 0.455423 | 0.26436 | **162** | 0.768657 | 0.517327 |
| **28** | 0.366204 | 0.731971 | **73** | 0.534842 | 0.273851 | **118** | 0.711869 | 0.631726 | **163** | 0.595764 | 0.641142 |
| **29** | 0.733636 | 0.622361 | **74** | 0.20034 | 0.662925 | **119** | 0.855389 | 0.475627 | **164** | 0.827901 | 0.461475 |
| **30** | 0.305541 | 0.59079 | **75** | 0.512866 | 0.449958 | **120** | 0.546061 | 0.518807 | **165** | 0.422616 | 0.638589 |
| **31** | 0.463157 | 0.688255 | **76** | 0.497052 | 0.694409 | **121** | 0.171889 | 0.164067 | **166** | 0.89756 | 0.610079 |
| **32** | 0.13226 | 0.853682 | **77** | 0.130588 | 0.519482 | **122** | 0.36096 | 0.418281 | **167** | 0.839559 | 0.609484 |
| **33** | 0.308964 | 0.58721 | **78** | 0.495431 | 0.329144 | **123** | 0.085568 | 0.630105 | **168** | 0.551696 | 0.77115 |
| **34** | 0.203147 | 0.655741 | **79** | 0.504045 | 0.576343 | **124** | 0.575276 | 0.699216 | **169** | -0.00451 | 0.431929 |
| **35** | 0.692814 | 0.473939 | **80** | 0.733734 | 0.717806 | **125** | 0.472748 | 0.532929 | **170** | 0.349746 | 0.642216 |
| **36** | 0.587298 | 0.622751 | **81** | 0.409822 | 0.208159 | **126** | 0.650251 | 0.717714 | **171** | 0.472682 | 0.481767 |
| **37** | 0.469409 | 0.296074 | **82** | 0.384826 | 0.79145 | **127** | 0.631646 | 0.463007 | **172** | 0.359802 | 0.696045 |
| **38** | 0.699784 | 0.662233 | **83** | 0.158699 | -0.1197 | **128** | -0.00017 | 0.533796 | **173** | 0.68085 | 0.169557 |
| **39** | 0.55323 | 0.334659 | **84** | 0.37233 | 0.348738 | **129** | 0.290512 | 0.5159 | **174** | 0.329336 | 0.545563 |
| **40** | -0.15051 | 0.530129 | **85** | 0.557595 | 0.234802 | **130** | 0.629574 | 0.321096 | **175** | -0.20992 | -0.02057 |
| **41** | -0.26437 | 0.696353 | **86** | 0.399819 | 0.267315 | **131** | 0.331694 | 0.572857 | **176** | -0.09157 | 0.180922 |
| **42** | 0.543016 | 0.494554 | **87** | 0.61902 | 0.495224 | **132** | 0.069993 | 0.4507 | **177** | 0.223775 | 0.487016 |
| **43** | 0.117034 | 0.656485 | **88** | 0.579132 | 0.604864 | **133** | 0.828536 | 0.696427 | **178** | 0.698964 | 0.270185 |
| **44** | 0.361109 | 0.631241 | **89** | 0.657888 | 0.496978 | **134** | -0.15983 | 0.449546 | **179** | 0.583331 | 0.58418 |
| **45** | 0.763765 | 0.637221 | **90** | 0.56696 | 0.618335 | **135** | 0.165899 | 0.803282 | **180** | 0.664383 | 0.426401 |

**Table S8.** ICC values per image across sessions 1 and 5 for the full set of 180 images.

| *Natural-cued condition* | | | | | |
|---|---|---|---|---|---|
| **Parameters** | **session 1** | **session 2** | **session 3** | **session 4** | **session 5** |
| **Drift bias** | $0.021 \pm 0.079$ | $-0.029 \pm 0.097$ | $0.02 \pm 0.106$ | $0.082 \pm 0.171$ | $-0.036 \pm 0.125$ |
| **Health weight** | $0.871 \pm 0.475$ | $0.671 \pm 0.296$ | $0.719 \pm 0.31$ | $0.709 \pm 0.39$ | $0.838 \pm 0.483$ |
| **Taste weight** | $-0.05 \pm 0.278$ | $0.084 \pm 0.472$ | $0.041 \pm 0.475$ | $0.005 \pm 0.608$ | $0.065 \pm 0.45$ |
| **NDT** | $0.599 \pm 0.163$ | $0.595 \pm 0.094$ | $0.58 \pm 0.15$ | $0.54 \pm 0.115$ | $0.552 \pm 0.089$ |
| **SP bias** | $0.492 \pm 0.035$ | $0.501 \pm 0.035$ | $0.5 \pm 0.035$ | $0.481 \pm 0.038$ | $0.504 \pm 0.042$ |
| **Noise** | $1.017 \pm 0.129$ | $1.023 \pm 0.141$ | $1.08 \pm 0.134$ | $1.08 \pm 0.16$ | $1.093 \pm 0.123$ |
| *Health-cued condition* | | | | | |
| **Parameters** | **session 1** | **session 2** | **session 3** | **session 4** | **session 5** |
| **Drift bias** | $0.047 \pm 0.074$ | $0.074 \pm 0.132$ | $0.012 \pm 0.119$ | $0.085 \pm 0.072$ | $-0.056 \pm 0.086$ |
| **Health weight** | $0.322 \pm 0.321$ | $0.288 \pm 0.225$ | $0.303 \pm 0.289$ | $0.33 \pm 0.313$ | $0.346 \pm 0.423$ |
| **Taste weight** | $0.895 \pm 0.457$ | $0.82 \pm 0.553$ | $0.791 \pm 0.498$ | $0.669 \pm 0.652$ | $0.784 \pm 0.627$ |
| **NDT** | $0.588 \pm 0.13$ | $0.572 \pm 0.102$ | $0.526 \pm 0.154$ | $0.539 \pm 0.104$ | $0.516 \pm 0.096$ |
| **SP bias** | $0.49 \pm 0.035$ | $0.481 \pm 0.041$ | $0.489 \pm 0.038$ | $0.485 \pm 0.041$ | $0.508 \pm 0.036$ |
| **Noise** | $0.98 \pm 0.129$ | $1.007 \pm 0.119$ | $1.004 \pm 0.091$ | $1.078 \pm 0.123$ | $1.029 \pm 0.132$ |

**Table S9. Standard-DDM Parameters.** This table reports the group-level estimates (mean ± standard error (SE)) for each of the standard-DDM parameters over 5 sessions in both the natural-cued and health-cued conditions. The parameters are: stochastic component of evidence accumulation (*noise*), starting point bias (*SP bias*; a value of 0.5 indicate no starting point bias), non-decision time ($NDT$), weight of taste attribute on drift rate ($taste$), weight of taste attribute on drift rate ($health$), and intercept for drift rate ($Drift\ bias$). The decision thresholds were held fixed at [-1, 1] when fitting the model.

| Parameter | Natural-cued Condition | Health-cued Condition | Health - Natural |
|---|---|---|---|
| **Drift bias** | -0.0146 | -0.067 | -0.13 |
| **Health weight** | 0.538 | 0.718 | 0.541 |
| **Taste weight** | 0.671 | 0.838 | 0.808 |
| **NDT** | 0.446 | 0.625 | 0.0278 |
| **SP bias** | 0.00853 | -0.037 | -0.0248 |
| **Noise** | 0.519 | 0.479 | -0.0114 |

**Table S10. Intra-class correlation coefficients (ICC).** ICC of Standard-DDM parameters over 5 sessions for both conditions of natural-cued and health-cued and also for the difference between these two conditions. NDT = non-decision time.
